# The cognition, information behaviors, and preventive behaviors of Taiwanese people facing COVID-19

**DOI:** 10.1038/s41598-022-20312-6

**Published:** 2022-10-08

**Authors:** Hsiu-Ping Yueh, Guan-Yun Wang, Tony Szu-Hsien Lee

**Affiliations:** 1 Department of Psychology, National Taiwan University, Taipei, Taiwan; 2 Department of Bio-Industry Communication and Development, National Taiwan University, Taipei, Taiwan; 3 Graduate School of Information Science, Tohoku University, Sendai, 980-8577 Japan; 4 Department of Health Promotion and Health Education, National Taiwan Normal University, Taipei, Taiwan; 5 Continuing Education Master’s Program of Addiction Prevention and Treatment, National Taiwan Normal University, Taipei, Taiwan

**Keywords:** Human behaviour, Psychology

## Abstract

This study investigated the cognition, information behaviors and preventive behaviors of Taiwanese citizens in the face of the COVID-19 pandemic. An online survey was administered and 610 valid responses were collected. The relationships between demographic variables and optimistic bias, social trust, information credibility, personal protective measures, avoidance of human contact, and immune system strengthening were examined. Results showed that optimistic bias existed, but there was no significant correlation between optimistic bias and personal protective measures. Laypersons had high trust in the government, but also optimistic bias. Gender was the most important predictor; with occupation and region of residence also interacting with different preventive behaviors. People in Taiwan may be overly optimistic in facing the epidemic; relevant information should be properly disclosed to help reduce this bias. Social trust in the government seems to be an important successful factor in the fight against COVID-19 in Taiwan.

## Introduction

At the end of 2019, an unknown strain of pneumonia broke out; SARS-CoV-2, a novel coronavirus, began dramatically spreading in China and other countries, causing a respiratory illness now known as COVID-19. The entire world has since been influenced by this virus. Nearly three years later, the number of confirmed cases continues to rise rapidly^1,2^. People in Taiwan paid careful attention to this novel coronavirus from the very beginning, and the government of Taiwan quickly implemented strict policies^3^ so Taiwan initially had a period of relative stability to counteract the threat of COVID-19.

After Taiwan faced the SARS epidemic in 2003, Taiwan’s government built a complete system to handle contagious diseases of the lungs and respiratory tract that included confirmed case reporting, isolation and quarantine standards, and other precautions. When Taiwan’s health authorities first suspected the similarity of COVID-19 to the SARS virus, many policies, such as when to lock down a city were already encoded in the law. Taiwan’s government announced on December 31, 2019, that all passengers on airplanes arriving from Wuhan Province, China should be quarantined after entering Taiwan^4^.

To safeguard their health, people can employ proper health behaviors and take precautions to prevent infection with a virus or any disease, and they can also watch for symptoms during a possible incubation period after exposure^5^. However, if people have an optimistic bias (the belief that one is less likely to experience negative events than other people are, and that it is more likely one will experience positive events^6–8^), they may ignore self-management measures for preventing transmission. Previous studies have shown that social trust, the degree to which the public trusts the government, is an important consideration in the decision to follow guidelines provided by government authorities. Furthermore, because people nowadays can also access information about COVID-19 from many other sources, the credibility of these alternative sources is an important issue as well. If every individual believes that they have a lower-than-average chance of experiencing negative events, then on the scale of an entire group, the optimistic bias results in a systematic error, and accordingly becomes unrealistic optimism^8^. Research has indicated that personal experience, perceived controllability, and stereotype salience can influence an optimistic bias about negative events^8^.

The optimistic bias occurs when people experience risk. The perception of the possibility of risk depends on the stability of a positive self-image, because people need to balance negative event experiences and positive self-experiences^9^. Evidence shows that some groups, such as young adults, may have a stronger optimistic bias and believe that they have a greater ability to control future events than older adults^10^. The optimistic bias is related to several issues in public health research, such as bird flu outbreaks^9^, prostate cancer screening^11^, the H1N1 Influenza Vaccine^12^, food safety issues^13^, and also COVID-19^14,15^.

Government policies and actions play an important role in containing infectious diseases. When people face events with potentially high risk and they lack sufficient information, they tend to rely on social trust to make decisions^12^. Hu and Yueh indicated that if governments and media actively use social media channels, such channels can facilitate information transparency and avoid information inequality; they can be used to disclose information to the public^16^. Accordingly, the Taiwan Center for Disease Control and the Central Epidemic Communication Center held daily press conferences to communicate with the public about the COVID-19 situation, and they also used an official channel on Line, an instant messaging application, to release information every day^3^. Taiwanese people frequently use this kind of Internet media to check the news about the pandemic^17^, so providing information in this manner might enhance public trust^18^. The government also enacted strict policies to prevent the spread of rumors about COVID-19. COVID-19 information was conveyed primarily by the government, so social trust in the government was a key factor determining whether the government would be able to influence people’s attitudes to the disease.

When people face risk, they tend to search actively for related information and not to rely solely on the government^18–20^. Previous research that focused on information channels and sources^19,21,22^ indicated that because the government is an important information source, if it can enhance its credibility, the public’s optimistic bias will be reduced^16^. Authoritativeness and reputation are highly related to information credibility^23–25^. Because information with high credibility can reduce the uncertainty of information seekers, it makes them more willing to take related actions to overcome the risk^11^. During the spread of COVID-19, people were uncertain, so the information credibility of different sources likely resulted in different attitudes and behaviors.

**Figure 1:**
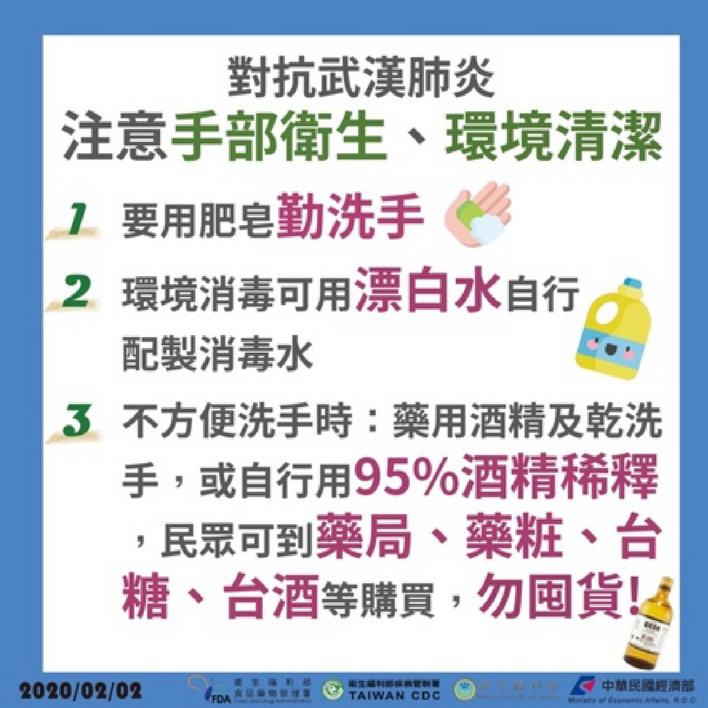
Promotional materials from the Ministry of Health and Welfare. Note: Translation: “Counteracting the Wuhan Virus: Pay attention to hand hygiene and environmental cleanliness. 1. Wash your hands frequently with soap. 2. Disinfect your surroundings with bleach. 3. When it is inconvenient to wash your hands, dry wash your hands with medicinal alcohol or use a 95% alcohol solution available in pharmacies, Taiwan Sugar, or TTL to dilute. Don’t stockpile.”

Fig. 1 shows some promotional materials from Taiwan’s Ministry of Health and Welfare. Their main message was to take simple protective measures such as washing hands, wearing masks, and using alcohol for disinfection. At the beginning of the pandemic, the authorities recommended simple preventive measures as there was no vaccine^26,27^. Some doctors also encouraged the public to bolster their immune systems to defend against the virus. Social distancing and avoiding human contact were also seen as effective preventive measures, so even at press conferences, television journalists maintained a safe distance from one another.

In sum, the purpose of this study is to explore the relationships among optimistic bias, social trust, information credibility, and preventive measures related to COVID-19, as well as to identify any behavioral differences according to gender, age, occupation, and region of residence. The results shed light on people’s cognitions and information-seeking and preventive behaviors and provide some referential information about Taiwan’s pandemic experience, especially during the initial key period.

## Materials and Methods

### Ethics and Participants

The Research Ethics Committee of National Taiwan Normal University reviewed and approved this protocol (No. 202002HS007). All methods were performed in accordance with the relevant guidelines and regulations. Respondents were recruited by convenience sampling mainly via social media and email advertisements. Minimum necessary sample size was calculated prior to the study using WebPower^28^, resulting in a recommendation of 341 participants to ensure statistical power greater than 0.8. In recruiting participants, special consideration was given to representation by gender, occupation, income, and area of residence using the snowball technique by inviting initial informants of various backgrounds. From February 27 to March 4, 2020, 709 participants voluntarily and anonymously filled out the online questionnaire without compensation. Informed consent was obtained from all subjects. Respondents older than 100 years and younger than 7 years were excluded, as were responses with decimals, blank responses, or errors. Those responses were regarded as invalid. A total of 610 valid responses were retained.

Of the 610 respondents, 119 were male and 491 were female; the average age was 44.01 years (range 11–71). See Table 1 for demographic details. There were many more female than male participants in this study, which is consistent with the common gender effect in questionnaire responses reported in the past literature^29^. In general, females are much more likely to actively participate in a survey than males.

**Table 1:** Demographic variables (*n* = 610).

Items	*n*	%
**Gender**		
Male	119	19.51%
Female	491	80.49%
**Region**		
Northern Taiwan	415	68.03%
Other regions	195	31.97%
**Age**		
Under 30	93	15.25%
31 ~ 40	137	22.46%
41 ~ 50	208	34.10%
Above 51	172	28.20%
**Education**		
Undergraduate	332	54.43%
Graduate School	278	45.57%
**Occupation**		
Government employee	220	36.07%
Healthcare	100	16.39%
Other	290	47.54%
**Average monthly income**		
Less than 50,000	316	51.80%
Over 50,000	294	48.20%

### Measures

#### Optimistic bias

There are two methods for measuring the optimistic bias: relative measures and absolute measures. This research chose to use an absolute measure–the possibility of personal risk and the possibility of other people experiencing risk were estimated separately^30^. Participants responded to at least these two variants of each item. To measure participants optimistic bias, three items modified from Lu, et al.^19^, Lu, et al.^31^, Weng, et al.^32^, were added “You think you yourself may be infected with COVID-19”, “You think your neighbors or colleagues may be infected with COVID-19” and “You think others may be infected by COVID-19”. These items were scored on a six-point Likert-type scale (1 = *strongly disagree* to 6 = *strongly agree*). This study first examined whether significant differences between these three items existed, and then used the score of Others minus Self to calculate the level of optimistic bias. The higher the score, the greater the optimistic bias is.

#### Social trust

To measure social trust, this study modified Wang and Yueh’s scale^13^, which consists of four items: “I think the government is credible to enact policies on COVID-19”, “I think the government is correct to enact policies on COVID-19”, “I think the government should develop long-term plans for COVID-19”, and “I think the government can solve problems related to COVID-19.” The scale employed a six-point Likert-type scale (1 = *strongly disagree* to 6 = *strongly agree*).

#### Information credibility

To measure information creditability, this study modified Wang and Yueh^13^ and Lu, et al.'s information credibility scales^31^. They consist of five items rated on a six-point Likert-type scale (1 = *strongly disagree* to 6 = *strongly agree*), including “Information about COVID-19 from family members and friends is credible”, “Information about COVID-19 from newspapers, television and radio is credible”, “Information about COVID-19 on the Internet and social media is credible”, “Information about COVID-19 from research institutes is credible” and “Information about COVID-19 from the government is credible.”

#### Protective measures

##### Personal protective measures

Personal protective measures included “wear a mask”, “take eye protection measures”, “wash your hands frequently with soap”, and “avoid touching your eyes, nose, and mouth”. This scale used a 4-point scale from 1 = *rarely* to 4 = *always* to evaluate the frequency of engaging in these behaviors.

##### Avoiding human contact

Behaviors for avoiding human contact included “avoid close contact with other people”, “avoid crowded places”, “when I feel ill, I distance myself from others”, “if you feel ill, immediately notifying the person in charge of the epidemic, such as a doctor or a neighbor”, “avoid taking public transportation”, “avoid entering physical shops, and shop online instead”, and “avoid unnecessary travel”. This scale was also rated on a 4-point scale from 1 = *rarely* to 4 = *always* to evaluate the frequency of engaging in these behaviors.

##### Strengthening one’s immune system

Participants rated behaviors for strengthening their immune system on a 4-point scale from 1 = *rarely* to 4 = *always*. The scale consisted of seven items, which included “do more exercise”, “balance nutrition and consume nutritional supplements”, “keep positive emotions”, “sleep enough”, “drink more water”, “purchase masks and alcohol disinfectant”, and “buy tissue paper and wet napkins.”

#### Demographic variables

Respondents provided their demographic information including gender, personal monthly income, education, age, occupation, and region of residence.

## Results

### Descriptive statistics, reliability, and correlation

R 3.5.3 in Rstudio 1.1.414 was used to conduct the statistical analyses. The descriptive statistics and reliability results are listed in Table 2. Personal protective measures included wearing a mask, taking eye protection measures, washing your hands frequently with soap, and avoiding touching your eyes, nose, and mouth. Washing your hands frequently was the highest rated behavior, and taking eye protection measures was the lowest. The highest rated behaviors for avoiding human contact were distancing from others and avoiding unnecessary travel. Behaviors related to strengthening the immune system were scored lower than other types of behaviors, but them, drinking more water was the most highly rated.

**Table 2:** Descriptive statistics (*n* = 610).

Items	Mean	SD
**Optimistic bias** (1–6, strongly disagree to strongly agree) (α = 0.85)		
You think you may be infected by COVID-19	3.34	0.90
You think your neighbors or colleagues may be infected by COVID-19	3.53	0.80
You think others may be infected by COVID-19	3.96	0.80
**Social Trust** (1–6, strongly disagree to strongly agree) (α = 0.91)		
I think the government is credible to adopt policy on COVID-19	4.77	1.01
I think the government is correct to adopt policy on COVID-19	4.73	1.01
I think the government should develop long-term plans for COVID-19	5.05	0.92
I think the government can solve problems related to COVID-19	4.67	1.01
**Information Credibility** (1–6, strongly disagree to strongly agree) (α = 0.77)		
Information about COVID-19 from family members and friends is credible	3.57	0.86
Information about COVID-19 from newspapers, television and radio is credible	3.99	0.86
Information about COVID-19 on the Internet and social media is credible	3.55	0.87
Information about COVID-19 from research institutes is credible	4.68	0.89
Information about COVID-19 from the government is credible	4.84	1.00
**Personal Protective Measures** (1–4, rarely to always) (α = 0.66)		
Wear a mask	3.11	0.85
Take eye protection measures	2.22	1.08
Wash your hands frequently with soap	3.47	0.68
Avoid touching your eyes, nose, and mouth	3.11	0.82
**Avoiding Human Contact** (1–4, rarely to always) (α = 0.77)		
Avoid close contact with other people	3.07	0.79
Avoid crowded places	3.28	0.67
When I feel ill, I distance myself from others	3.59	0.57
If you feel ill, immediately notify the person in charge of the epidemic, such as a doctor or a neighbor	2.98	0.97
Avoid taking public transportation	2.90	0.98
Avoid entering physical shops and shop online instead	2.69	0.95
Avoid unnecessary travel	3.57	0.72
**Strengthening One’s Immune System** (1–4, rarely to always) (α = 0.73)		
Do more exercise	2.17	0.58
Balance nutrition and consume nutritional supplements	2.32	0.58
Keep positive emotions	2.18	0.58
Sleep enough	2.22	0.60
Drink more water	2.40	0.64
Purchase masks and alcohol disinfectant	2.30	0.92
Buy tissue paper and wet napkins	2.10	0.66

Variable correlations are listed in Table 3. The correlation table shows a positive correlation between participants’ optimistic bias and immune system strengthening, as well as positive correlations between social trust and information credibility, personal protective behavior, and avoiding human contact. Information credibility was positively correlated with contact avoidance and immune system strengthening behaviors. Avoiding human contact was positively correlated with strengthening one’s immune system.

**Table 3:** Correlations of all variables.

	OptimismBias	Social trust	Information credibility	Personal protective	Avoid contact	Improve immune
Optimistic bias	–	–	–	–	–	–
Social trust	0.0108	–	–	–	–	–
Information credibility	0.0412	0.6065***	–	–	–	–
Personal protective measures	−0.0622	0.0894*	0.0577	–	–	–
Avoid human contact	0.0028	0.1131**	0.1589***	0.3889***	–	–
Improve immune system	0.0885*	0.0129	0.0852***	0.1515***	0.1094***	–

*p < .05, **p < .01, ***p < .001.

### Test of the optimistic bias

**Figure 2:**
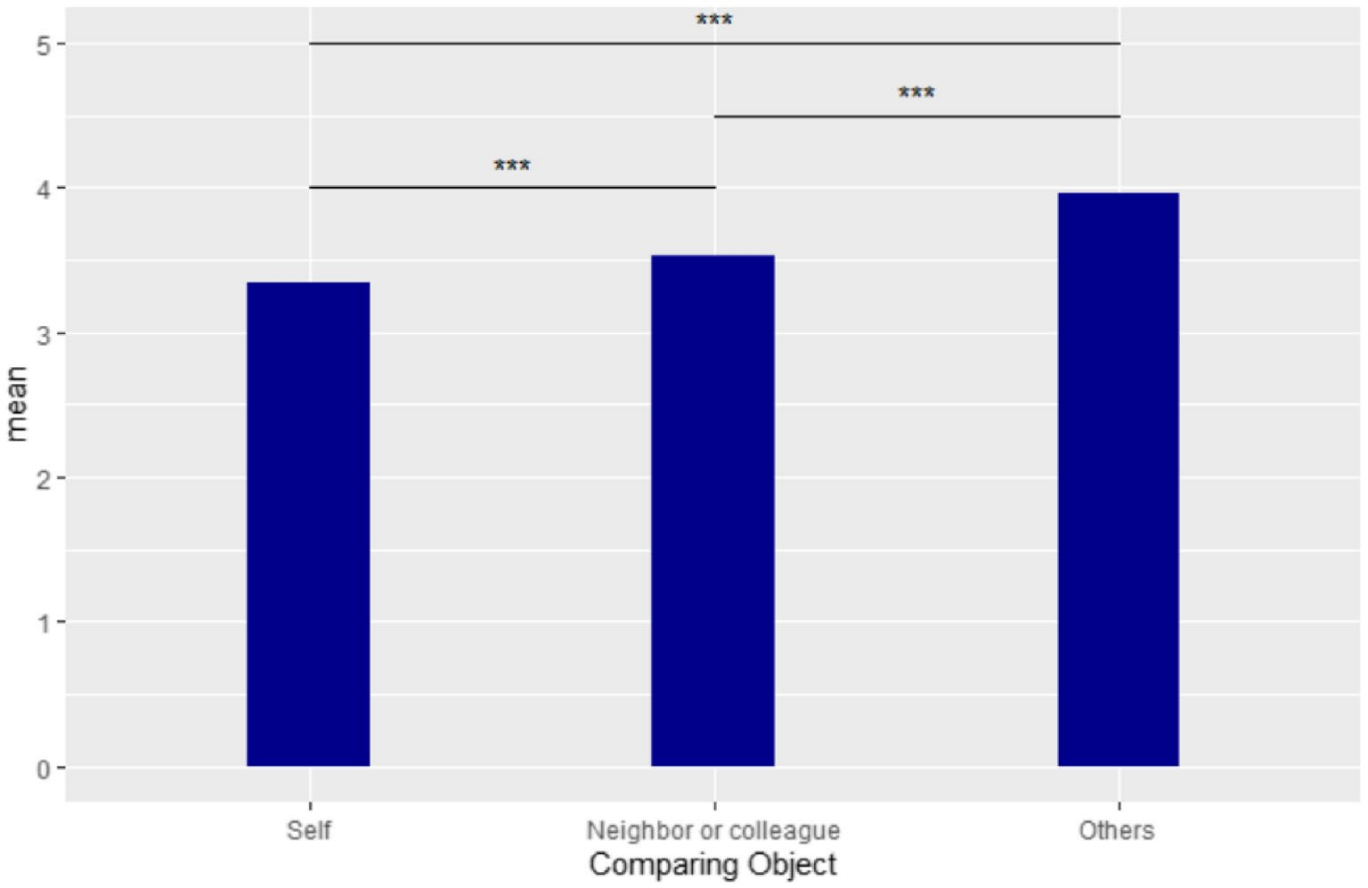
Optimistic bias scores. Note: ***p < .001.

A *t*-test indicated a significant difference between participants’ perception of risk for self and others. The difference between these two variables is the degree of optimistic bias. Paired *t*-tests showed that the item “you think your neighbors or colleagues may be infected by COVID-19” (M = 3.53) was significantly higher than the item “you think you yourself may be infected by COVID-19” (M = 3.34)(t = −8.2092, *df* = 609, *p* < 0.001), and the item “you think others may be infected by COVID-19” (M = 3.96) was significantly higher than the item “you think you yourself may be infected by COVID-19” (M = 3.34)(*t* = −18.21, *df* = 609, *p* < 0.001). In addition, the perception that others may be infected was also significantly higher than perceptions of neighbors or colleagues (*t* = −15.957, *df* = 609, *p* < 0.001). These results indicate that the individual members of a group thought that they themselves were less likely to be infected, which indicates a systematic optimistic bias error. It also suggests that people not in one’s daily life were regarded as more likely to be infected than those within one’s social circle. Fig. 2 shows the relationships among these three risk perceptions.

### Comparison of channel information credibility

**Figure 3:**
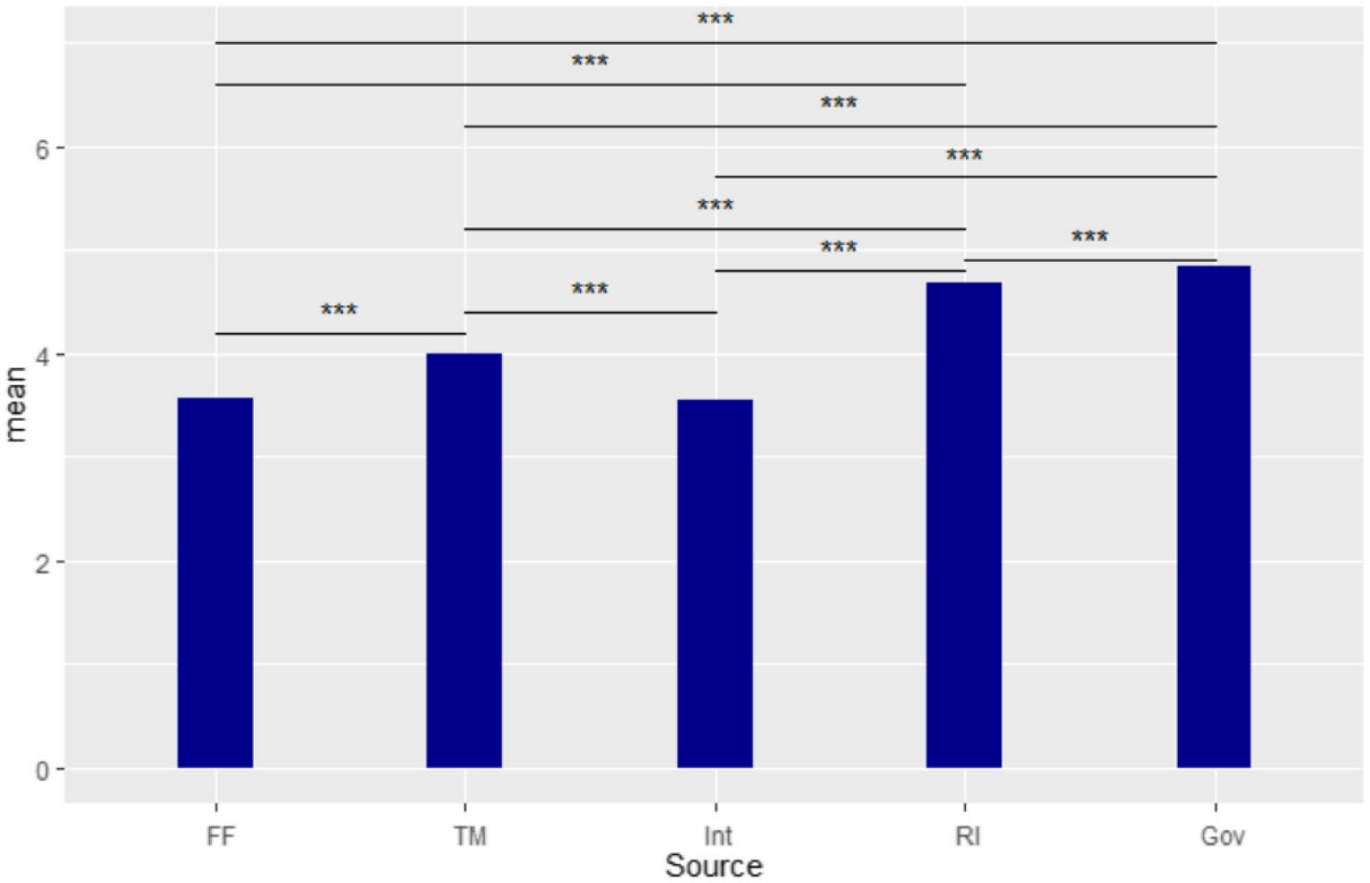
Information Credibility Comparisons. Note: ***p < .001. FF = family members and friends; TM = traditional media; Int = the Internet and social media; RI = research institutes; Gov = government.

A repeated measures ANOVA was performed to test the credibility of the different information sources. The same participant gave a credibility score to each of the sources of information provided in the questionnaire. The results showed significant differences in credibility between different sources of information (F(4,609) = 465.9965, *p* < 0.001). The results of multiple comparisons by Tukey’s method are listed in Table 4. Information from the government and research institutes had higher credibility than that from other sources, with the government having the highest score (See Table 4 and Fig. 3).

**Table 4:** Comparison of credibility of different information by Tukey's method.

Comparison	estimate	*df*	*t* ratio	F
Family members and friends—Newspapers, television and radio	−0.4229	2436	−10.6163	< 0.0001
Family members and friends—The Internet and social media	0.02295	2436	0.5760	0.9785
Family members and friends—Research institutes	−1.1098	2436	−27.8576	< 0.0001
Family members and friends—Government	−1.2704	2436	−31.8902	< 0.0001
Newspapers, television and radio—The Internet and social media	0.4459	2436	11.1924	< 0.0001
Newspapers, television and radio—Research institutes	−0.6868	2436	−17.2413	< 0.0001
Newspapers, television and radio—Government	−0.8475	2436	−21.2738	< 0.0001
The Internet and social media—Research institutes	−1.1327	2436	−28.4337	< 0.0001
The Internet and social media—Government	−1.2934	2436	−32.4662	< 0.0001
Research institutes – Government	−0.1606	2436	−4.03256	0.0005

### Four-way ANOVA of key variables

Whether the optimistic bias corresponds to people's preventive behavior was examined with four important demographic variables: gender, age, occupation, and region of residence. It is expected that gender would reflect the influence prevention behaviors may have on preparedness behaviors, and that age may reflect different generations' ways of thinking and behaving. The occupations in the study were primarily divided between government, medical professions, and other professional workers all of which have different competencies and literacies in policy and public health. In addition, in the early 2020s, cases in northern Taiwan were much higher than elsewhere in Taiwan so residents in each region may have had different levels of perceived possible impact of the outbreak. Based on these considerations, the four demographic variables were considered independent variables in this study, and the degree of optimistic bias, social trust, information credibility, personal protective measures, avoidance of human contact, and strengthening of self-immunity were considered to be dependent variables.

#### Optimistic bias

**Figure 4:**
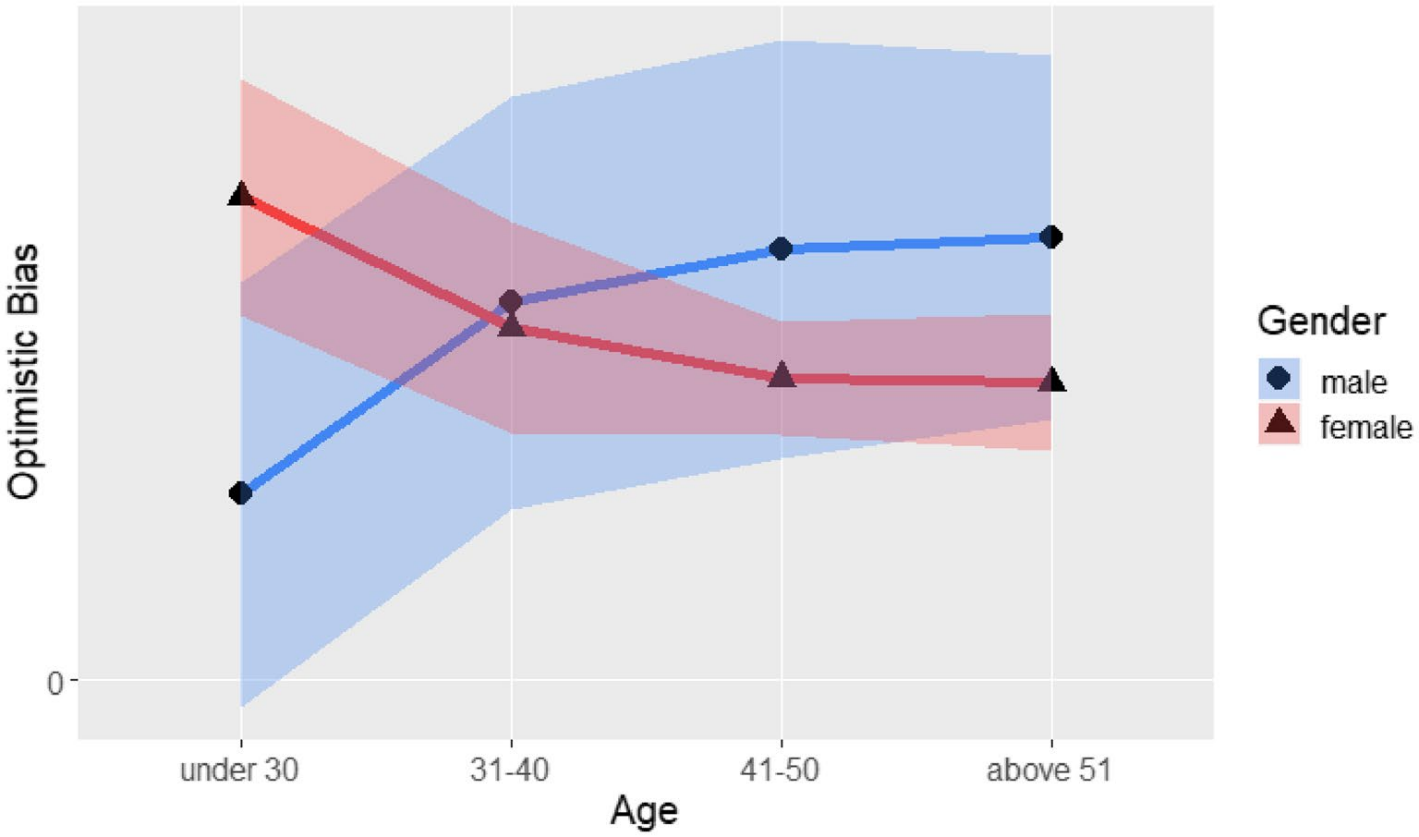
Optimistic bias score by gender and age.

Table 5 lists the ANOVA results of the optimistic bias by gender, region of residence, age, and occupation. The results indicated no significant four-way interaction effect and no significant three-way interaction effect, but there was a significant two-way interaction effect between gender and age (F(3, 563) = 3.64, *p* < 0.05). As shown in Fig. 4, males and females had opposite trends. Older men and younger women were associated with greater optimistic bias scores. There was also a significant main effect for occupation (F(2, 563) = 4.17, *p* < 0.05). The main effect test by Tukey’s method showed that people who worked in health care industries had less optimistic bias than did those in other occupations (*t*(563) =  − 2.37, *p* < 0.05).

**Table 5:** Four-way analysis of variance of optimistic bias with gender, region, age and occupation.

Source	SS	df	MS	F	Sig
Gender	0.3556	1	0.3556	0.5034	0.4783
Region	0.0111	1	0.0111	0.0156	0.9005
Age	1.3705	3	0.4568	0.6467	0.5853
Occupation	5.9289	2	2.9645	4.1962	0.0155*
Gender × Region	0.0802	1	0.0802	0.1135	0.7364
Gender × Age	7.7278	3	2.5759	3.6463	0.0126*
Region × Age	1.5275	3	0.5092	0.7207	0.5399
Gender × Occupation	0.7772	2	0.3886	0.5501	0.5772
Region × Occupation	1.4429	2	0.7214	1.0212	0.3608
Age × Occupation	2.3753	6	0.3959	0.5604	0.7619
Gender × Region × Age	1.8611	3	0.6204	0.8782	0.4521
Gender × Region × Occupation	2.8567	2	1.4283	2.0218	0.1334
Gender × Age × Occupation	4.6905	6	0.7817	1.1066	0.3570
Region × Age × Occupation	1.0775	6	0.1796	0.2542	0.9576
Gender × Region × Age × Occupation	5.9174	5	1.1835	1.6752	0.1386
Error	397.7351	563	0.7065		

* *p* < .05.

#### Social trust

**Figure 5:**
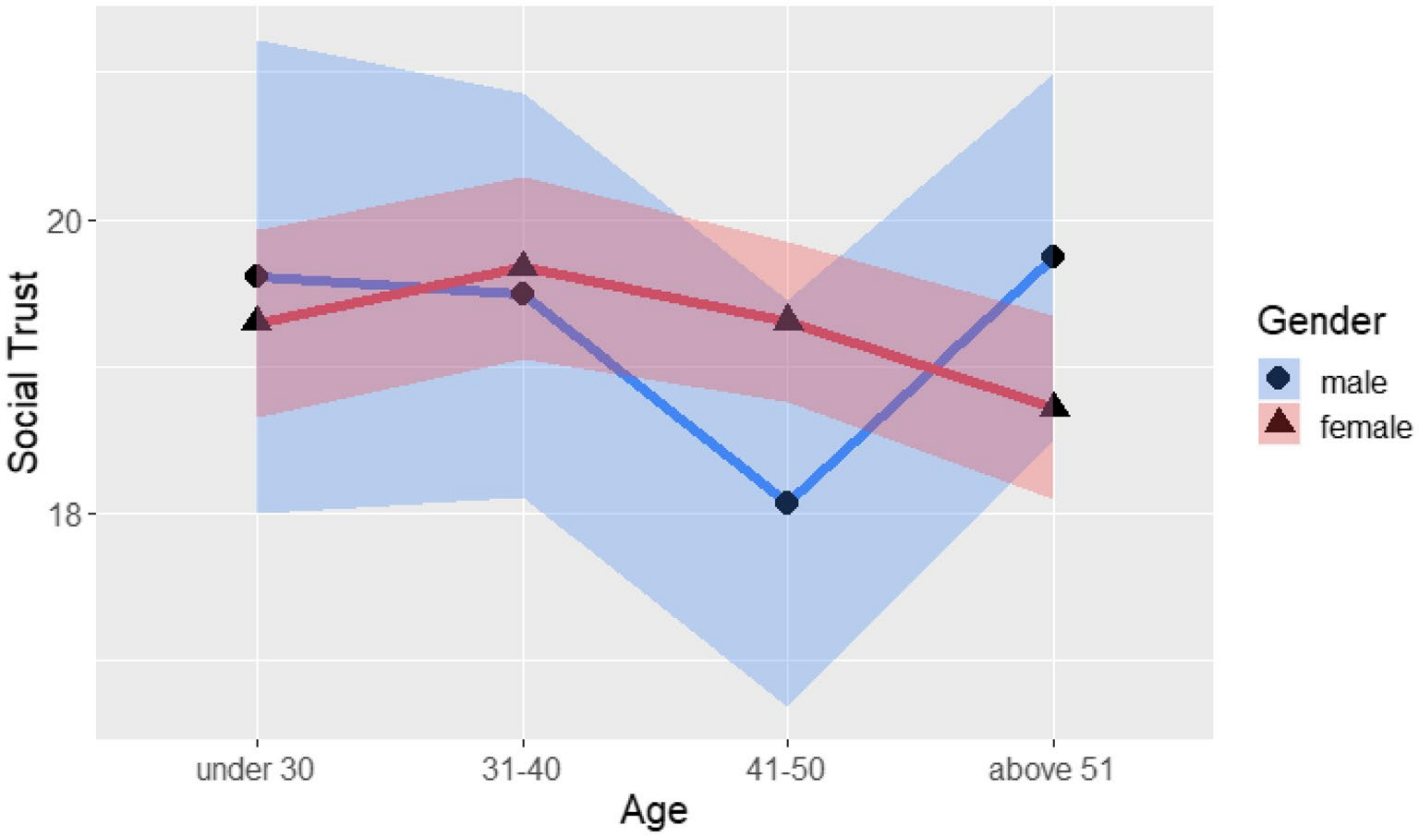
Social trust score with age and gender.

Table 6 presents the results of an ANOVA conducted with social trust as the dependent variable and the independent variables of gender (2 levels: male, female), region (2 levels: northern Taiwan, other regions of Taiwan), age (4 levels: under 30, 31–40, 41–50, above 51), and occupation (3 levels: government, healthcare industry, other). The results revealed no significant four-way interaction effect and no significant three-way interaction effect, but did identify a significant two-way interaction effect for gender and age: F(3, 563) = 2.65, *p* < 0.05, as shown in Fig. 5.

**Table 6:** Four-way analysis of variance of social trust with gender, region, age and occupation.

Source	SS	*df*	MS	F	Sig
Gender	0.0113	1	0.0113	0.0009	0.9759
Region	2.6758	1	2.6758	0.2153	0.6428
Age	36.9387	3	12.3129	0.9909	0.3967
Occupation	22.4806	2	11.2403	0.9045	0.4053
Gender × Region	4.2413	1	4.2413	0.3413	0.5593
Gender × Age	98.7624	3	32.9208	2.6492	0.0481*
Region × Age	63.7763	3	21.2588	1.7108	0.1637
Gender × Occupation	33.3509	2	16.6755	1.3419	0.2622
Region × Occupation	72.4166	2	36.2083	2.9138	0.0551
Age × Occupation	36.4645	6	6.0774	0.4891	0.8167
Gender × Region × Age	9.1134	3	3.0378	0.2445	0.8653
Gender × Region × Occupation	48.8820	2	24.4410	1.9668	0.1409
Gender × Age × Occupation	35.6417	6	5.9403	0.4780	0.8248
Region × Age × Occupation	62.6878	6	10.4480	0.8408	0.5387
Gender × Region × Age × Occupation	67.9109	5	13.5822	1.0930	0.3631
Error	6996.1501	563	12.4266		

*p < .05.

#### Information credibility

Table 7 lists the ANOVA results with the dependent variable of information credibility. No interaction effect or main effects were found for any of the variables.

**Table 7:** Four-way Analysis of information credibility with gender, region, age and occupation.

Source	SS	*df*	MS	F	Sig
Gender	0.7517	1	0.7517	0.0720	0.7886
Region	32.2239	1	32.2239	3.0862	0.0795
Age	33.8041	3	11.2680	1.0792	0.3573
Occupation	6.3518	2	3.1759	0.3042	0.7379
Gender × Region	11.9398	1	11.9398	1.1435	0.2854
Gender × Age	64.9240	3	21.6413	2.0727	0.1028
Region × Age	54.3532	3	18.1177	1.7352	0.1587
Gender × Occupation	4.9742	2	2.4871	0.2382	0.7881
Region × Occupation	35.7601	2	17.8801	1.7124	0.1814
Age × Occupation	40.7660	6	6.7943	0.6507	0.6896
Gender × Region × Age	14.0950	3	4.6983	0.4500	0.7174
Gender × Region × Occupation	30.9972	2	15.4986	1.4843	0.2275
Gender × Age × Occupation	109.3296	6	18.2216	1.7451	0.1084
Region × Age × Occupation	21.6841	6	3.6140	0.3461	0.9122
Gender × Region × Age × Occupation	74.2431	5	14.8486	1.4221	0.2144
Error	5878.4794	563	10.4413		

#### Personal protective measures

**Figure 6:**
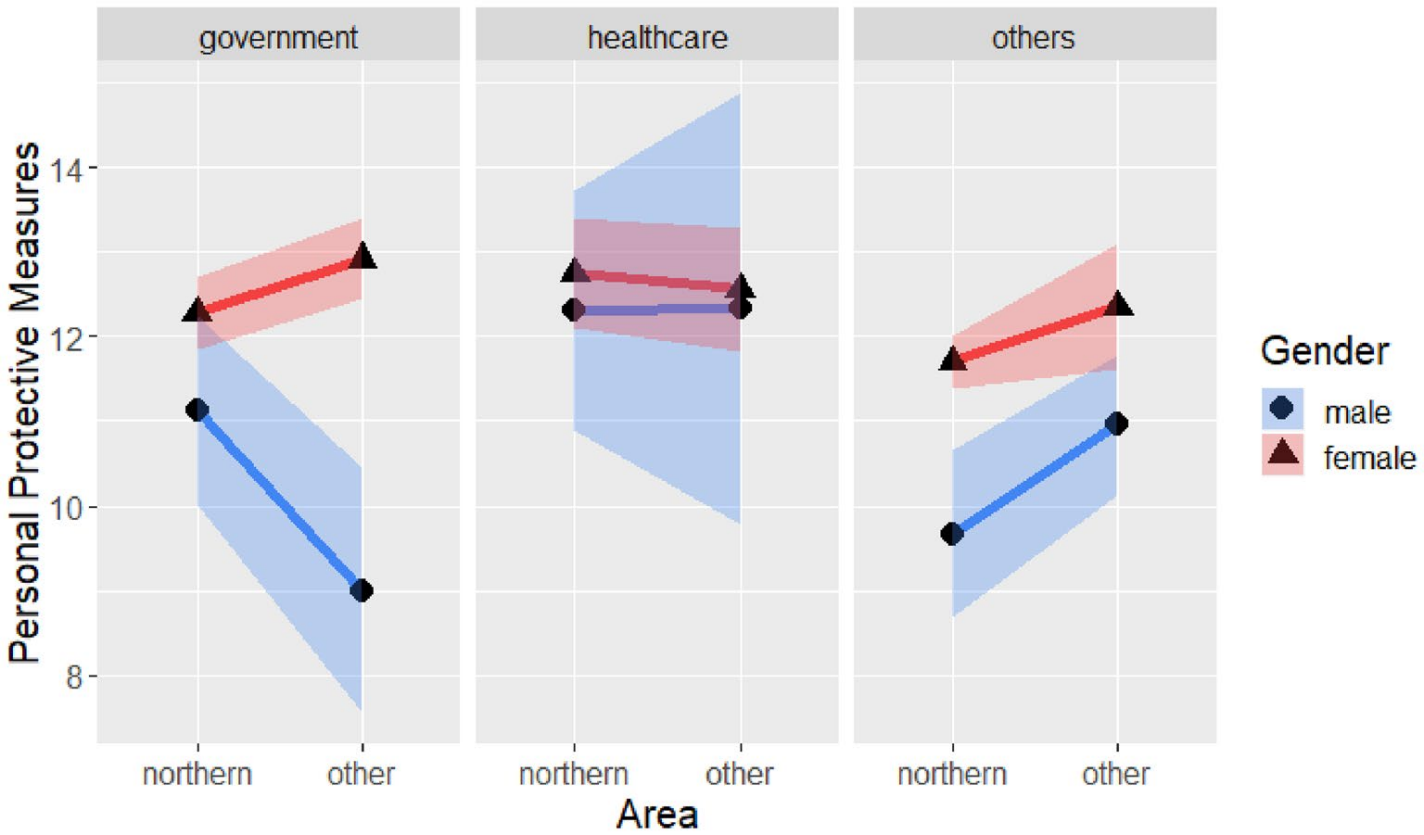
Three-way interaction of personal protective measures with gender, region and occupation.

The results of the ANOVA for personal protective measures with the four demographic variables are shown in Table 8. The results indicated a significant three-way interaction of gender, region of residence, and occupation: F(2, 563) = 5.47, *p* < 0.01, as shown in Fig. 6. The simple interaction effect was revealed (F(1, 216) = 58.55, *p* < 0.001) for government employees (see Table 9), Interaction effect of gender (see Table 10) was significant: male government employees in northern Taiwan took fewer personal protective measures than did their female colleagues (*t*(216) = −2.23, *p* < 0.05). In the other region category, all males took fewer personal protective measures than did females (*t*(216) = −3.92, *p* < 0.001). There was no significant interaction effect for healthcare industry employees, nor for those in the other occupation category.

**Table 8:** Four-way analysis of variance of personal protective measures with gender, region, age, and occupation.

Source	SS	df	MS	F	Sig
Gender	258.8366	1	258.8366	49.3845	0.0000***
Region	16.7981	1	16.7981	3.2050	0.0740
Age	14.6923	3	4.8974	0.9344	0.4237
Occupation	82.7593	2	41.3797	7.8950	0.0004***
Gender × Region	6.7784	1	6.7784	1.2933	0.2559
Gender × Age	7.8969	3	2.6323	0.5022	0.6809
Region × Age	20.4757	3	6.8252	1.3022	0.2728
Gender × Occupation	40.4085	2	20.2042	3.8548	0.0217*
Region × Occupation	26.3572	2	13.1786	2.5144	0.0818
Age × Occupation	8.9878	6	1.4980	0.2858	0.9437
Gender × Region × Age	35.0792	3	11.6931	2.2310	0.0836
Gender × Region × Occupation	57.3295	2	28.6648	5.4691	0.0044**
Gender × Age × Occupation	7.2365	6	1.2061	0.2301	0.9668
Region × Age × Occupation	16.3776	6	2.7296	0.5208	0.7927
Gender × Region × Age × Occupation	14.1275	5	2.8255	0.5391	0.7467
Error	2950.8244	563	5.2413		

*p < .05, **p < .01, ***p < .001.

**Table 9:** Simple interaction effect of different occupations and personal protective measures.

Source	SS	df	MS	F	Sig
**Government employee**					
Gender	164.08	1	164.083	31.9701	0.0000***
Region	1.55	1	1.546	0.3013	0.5836
Gender × Region	58.55	1	58.552	11.4084	0.0000***
Error	1108.60	216	5.132		
**Healthcare**					
Gender	2.24	1	2.2401	0.4415	0.5080
Region	0.48	1	0.4813	0.0948	0.7588
Gender × Region	0.15	1	0.1549	0.0305	0.8617
Error	487.12	96	5.0742		
**Others**					
Gender	131.12	1	131.1206	25.2931	0.0000***
Region	32.85	1	32.8547	6.3376	0.0123*
Gender × Region	3.80	1	3.8009	0.7331	0.3925
Error	1482.64	286	5.1840		

*p < .05, ***p < .001.

**Table 10:** Simple simple main effect of government employment on personal protective measures.

Source	SS	*df*	MS	F	Sig
**Government employees in Northern Taiwan**					
Gender	25.5187	1	25.5187	4.96967	0.0276*
Error	616.1862	120	5.1349		
**Government employees in other regions**					
Gender	194.7842	1	194.7842	37.9751	< 0.001***
Error	492.4096	96	5.1292		

*p < .05, ***p < .001.

**Figure 7:**
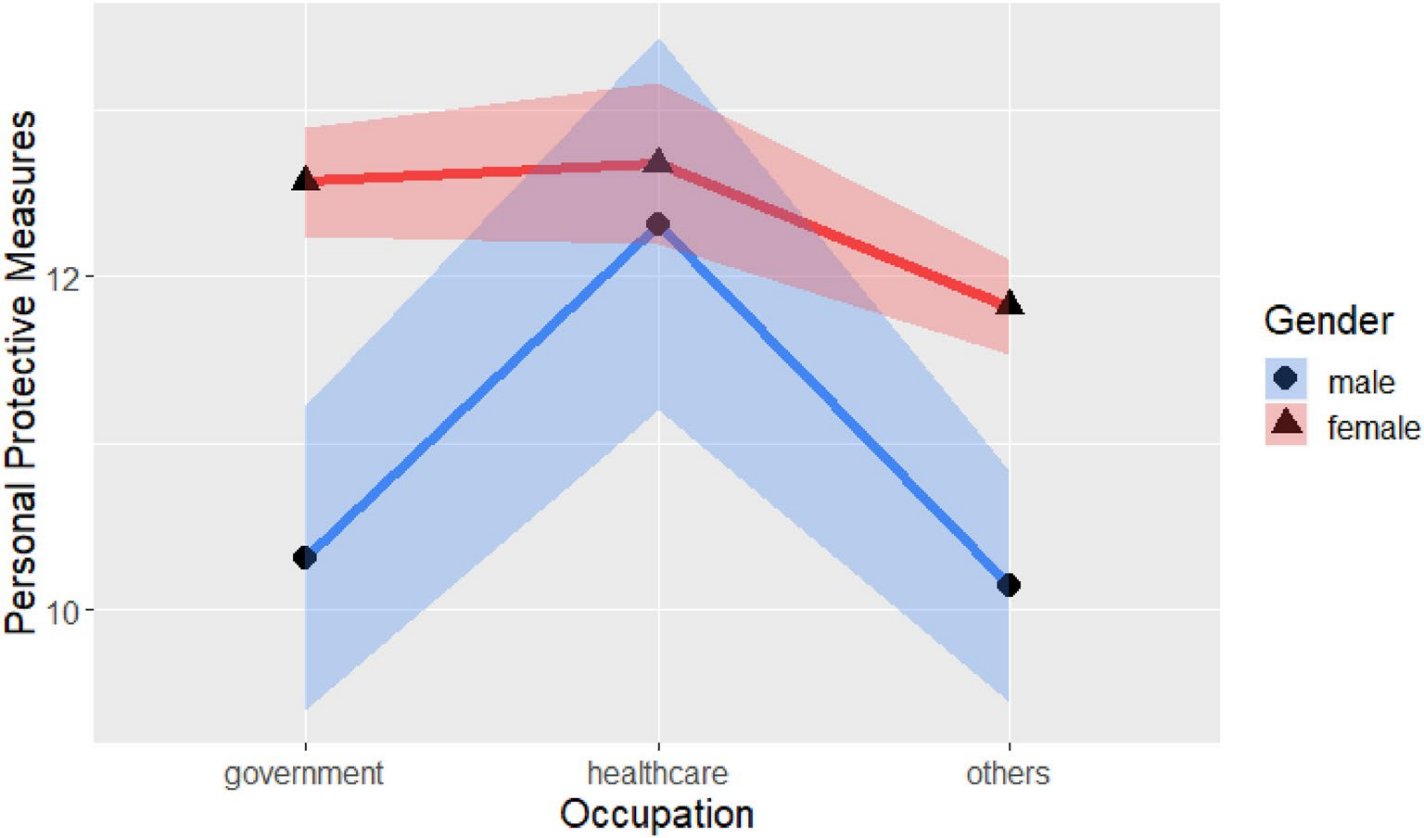
Personal protective measures with gender and occupations.

Table 8 shows the significant two-way interaction of gender and occupation, as shown in Fig. 7. Males tended to take fewer personal protective measures than females did when not employed in the healthcare industry. Due to the insufficient sample size, no main effect test could be conducted. Analysis of the sample from northern Taiwan suggested that in other occupations, males took significantly fewer personal protective measures than did females (*t*(563) = −5.10, *p* < 0.001).

Finally, there was a significant main effect of gender (F(1,563) = 49.38, *p* < 0.001). The analysis also focused on samples from northern Taiwan due to insufficient sample size in the other regions. Males scored significantly lower on personal protective measures than did females (*t*(563) = −3.04, *p* < 0.01). There was also a significant main effect of occupation (F(2, 563) = 7.90, *p* < 0.001), and Tukey’s comparison showed that government employees took more personal protective measures than did people in the other occupations category (*t*(563) = 1.25, *p* < 0.05), as did healthcare employees (*t*(563) = 4.06, *p* < 0.001).

#### Avoiding human contact

**Figure 8:**
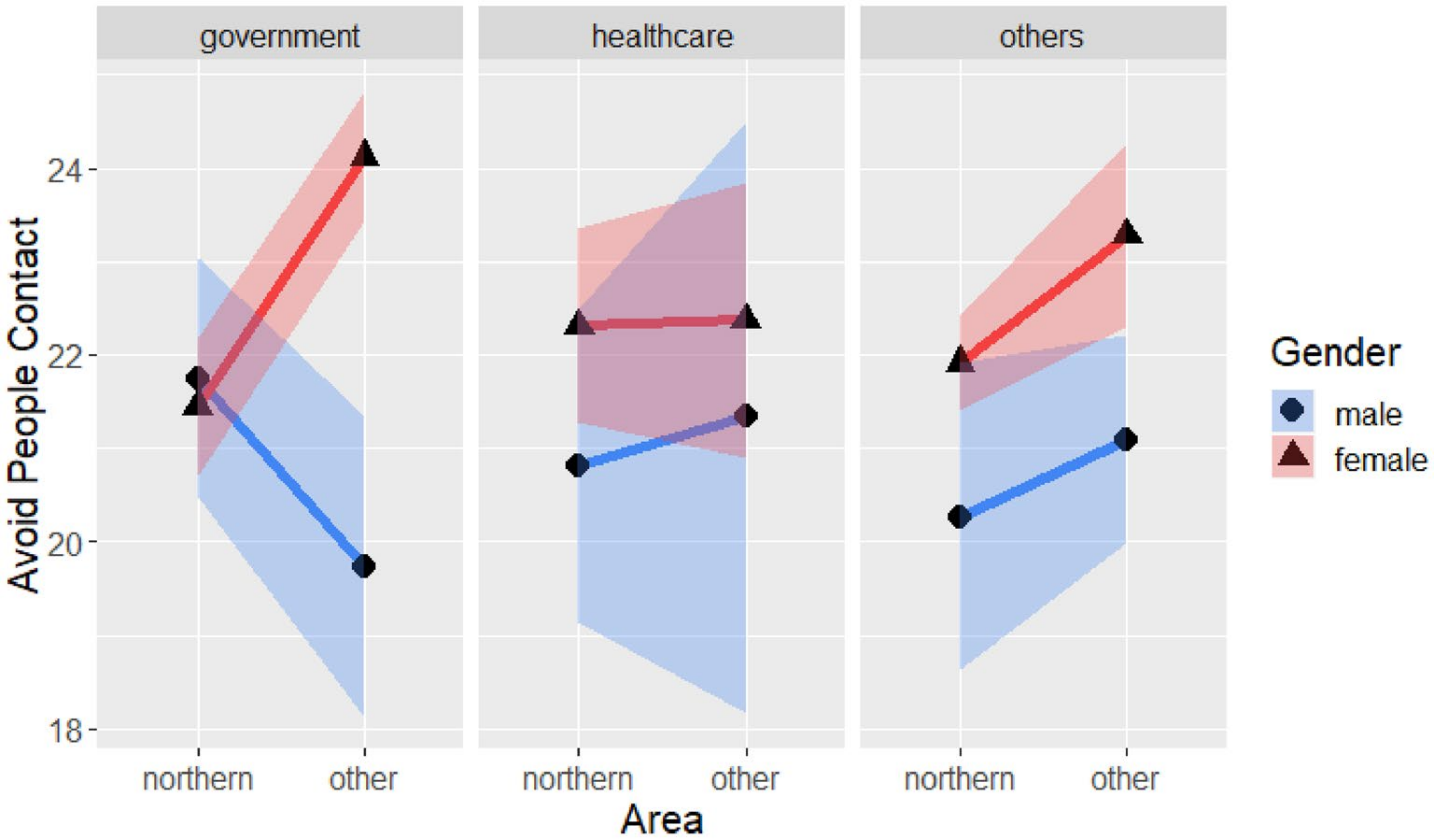
Three-way interaction of avoiding human contact with gender, region and occupation.

Table 11 lists the ANOVA results for avoiding human contact and the four demographic variables. No significant four-way interaction effect was found, but a significant three-way interaction effect was identified (F(2, 563) = 4.24, *p* < 0.05), as shown in Fig. 8. The simple interaction effect (Table 12) for government employees was significant (F(1, 216) = 14.84, *p* < 0.001). Accordingly, a simple main effect test was conducted (Table 13). It indicated that for government employees living in other regions of Taiwan males engaged in fewer behaviors for avoiding human contact than females (F(1,96) = 25.03, *p* < 0.001).

**Table 11:** Four-way analysis of variance of avoiding human contact with gender, region, age and occupation.

Source	SS	*df*	MS	F	Sig
Gender	244.0418	1	227.1508	19.8500	0.0000***
Region	227.1508	1	48.0932	18.4761	0.0000***
Age	144.2797	3	1.9971	3.9118	0.0088**
Occupation	3.9942	2	62.1975	0.1624	0.8501
Gender × Region	62.1975	1	21.3881	5.0591	0.0249*
Gender × Age	64.1644	3	5.2674	1.7397	0.1578
Region × Age	15.8021	3	6.5604	0.4284	0.7327
Gender × Occupation	13.1208	2	23.7139	0.5336	0.5868
Region × Occupation	47.4278	2	8.7063	1.9289	0.1463
Age × Occupation	52.2377	6	8.6786	0.7082	0.6432
Gender × Region × Age	26.0358	3	53.1953	0.7059	0.5488
Gender × Region × Occupation	106.3906	2	21.1563	4.3268	0.0137*
Gender × Age × Occupation	126.9377	6	11.4433	1.7208	0.1138
Region × Age × Occupation	68.6596	6	8.8087	0.9308	0.4722
Gender × Region × Age × Occupation	44.0435	5	12.2943	0.7165	0.6112
Error	6921.6863	563			

*p < .05, **p < .01, ***p < .001.

**Table 12:** Simple interaction effect of different occupations and avoiding human contact.

Source	SS	df	MS	F	Sig
**Government employee**					
Gender	92.6940	1	92.6941	8.1763	0.0046**
Region	189.9584	1	189.9584	16.7441	0.0000***
Gender × Region	168.3105	1	168.3105	14.8359	0.0001***
Error	2450.4734	216	11.3448		
**Healthcare**					
Gender	32.6221	1	32.6221	2.5357	0.1146
Region	0.4865	1	0.4864	0.0378	0.8462
Gender × Region	0.7539	1	0.7539	0.0586	0.8092
Error	1235.0475	96	12.8651		
**Others**					
Gender	118.656	1	118.6560	8.8967	0.0031**
Region	71.452	1	71.4518	5.3574	0.0213*
Gender × Region	2.813	1	2.8128	0.2109	0.6464
Error	3814.403	286	13.3371		

*p < .05, **p < .01, ***p < .001.

**Table 13:** Simple simple main effect of government employment on avoiding human contact.

Source	SS	df	MS	F	Sig
**Government employees in Northern Taiwan**					
Gender	1.7469	1	1.7469	0.13858	0.71036
Error	1512.7449	120	12.6062		
**Government employees in other regions**					
Gender	244.5164	1	244.5164	25.0324	0.0000***
Error	937.7285	96	9.7680		

***p < .001.

**Figure 9:**
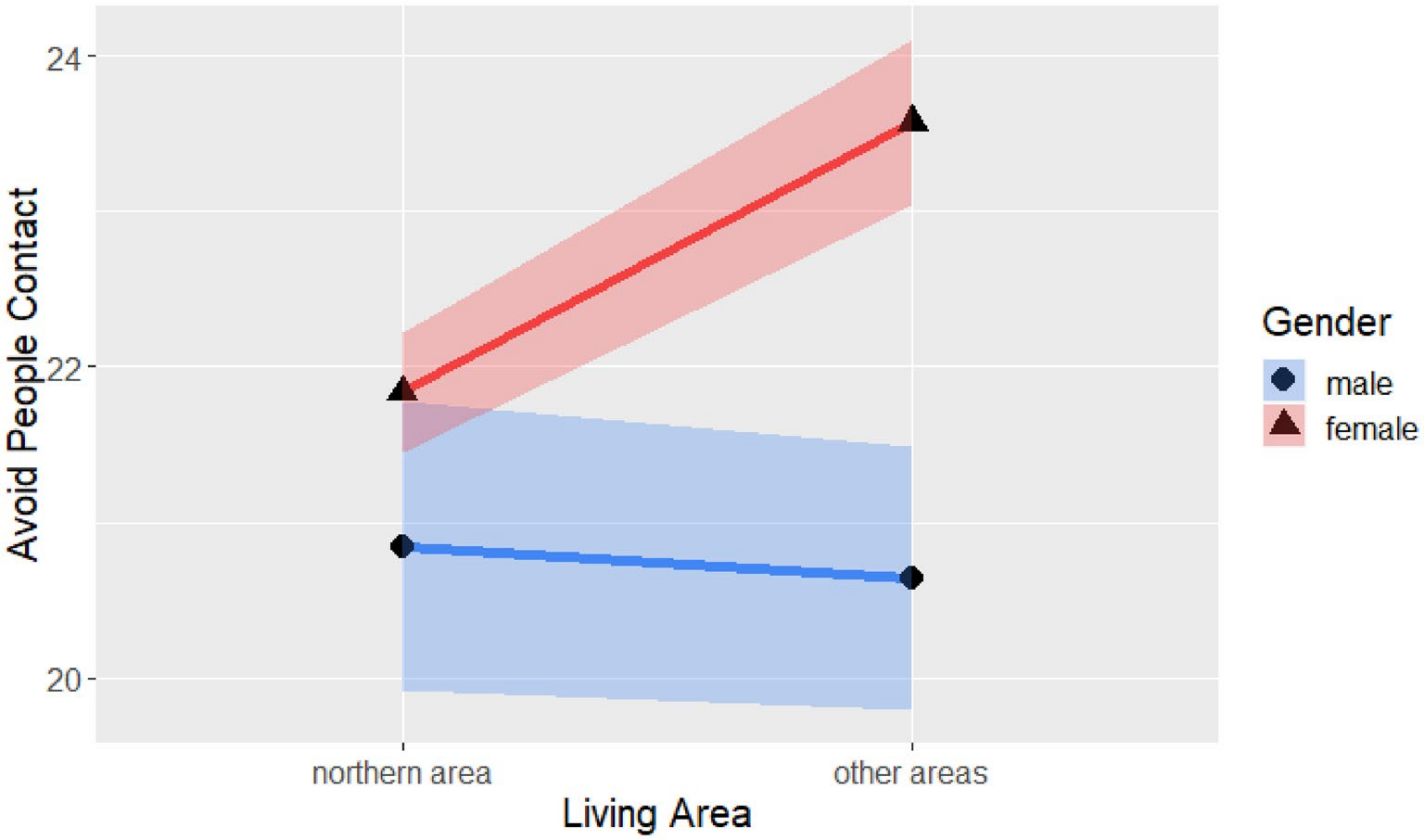
Avoiding human contact with gender and region.

There was also a significant two-way interaction of gender and region (F(1, 563) = 5.06, *p* < 0.05), as shown in Table 11 and Fig. 9, which suggests that people in the two regions avoided human contact to varying degrees according to their gender. A simple main effect test focused on the age group of 31–40 showed that males avoided human contact less than females did in both northern Taiwan (*t*(563) = −2.21, *p* < 0.05) and the other regions (t(563) = −2.76, *p* < 0.01).

Table 11 also shows three main effects. A significant main effect of gender (F(1, 563) = 19.85, *p* < 0.001) revealed that males engaged in avoidance behaviors less than females did. There was also a significant main effect of region (F(1, 563) = 18.48, *p* < 0.001); people in northern Taiwan avoided others less than did their counterparts in other regions. Furthermore, there was a significant main effect of age (F(1, 563) = 3.91, *p* < 0.01). Further comparison focused on females indicated that people under 30 avoided human contact less than adults aged 31–40 years did (*t*(563) = −3.32, *p* < 0.01).

#### Strengthening one’s immune system

**Figure 10:**
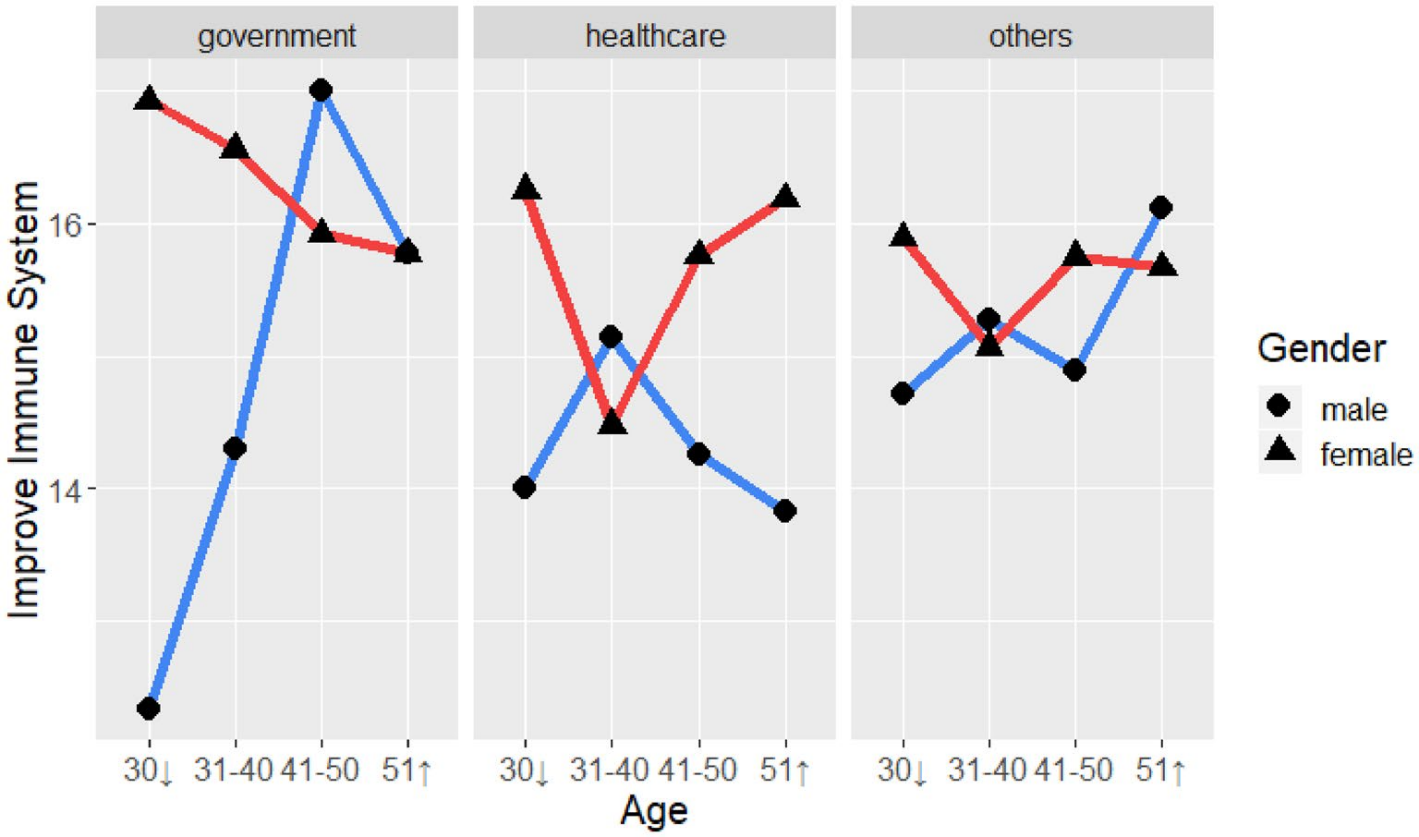
Three-way interaction of strengthening one’s immune system with gender and age for government employees.

Table 14 lists the ANOVA results for strengthening one’s immune system as a dependent variable with the four demographic variables. There was no significant four-way interaction, but there was a significant three-way interaction effect with gender, age, and occupation (F(6, 563) = 2.77, *p* < 0.05), as shown in Fig. 10. A further simple interaction test showed significant interactions of gender and age for government employees (F(3, 212) = 4.06, *p* < 0.01), which are listed in Table 15. As shown in Fig. 10, older male government employees compared to youngers tended to engage in more behaviors to strengthen their immune systems, but females maintained almost the same amount of such behaviors, but older females reduced them.

**Table 14:** Four-way analysis of variance of strengthening one’s immune system with gender, place and occupation.

Source	SS	df	MS	F	Sig
Gender	23.9029	1	8.6433	3.2222	0.0732
Region	8.6433	1	5.8879	1.1651	0.2809
Age	17.6638	3	21.1526	0.7937	0.4977
Occupation	42.3053	2	1.2653	2.8515	0.0586
Gender × Region	1.2653	1	15.0869	0.1706	0.6798
Gender × Age	45.2608	3	5.9392	2.0338	0.1081
Region × Age	17.8175	3	12.1479	0.8006	0.4938
Gender × Occupation	24.2958	2	1.9697	1.6376	0.1954
Region × Occupation	3.9393	2	2.1571	0.2655	0.7669
Age × Occupation	12.9425	6	16.8709	0.2908	0.9413
Gender × Region × Age	50.6126	3	7.8952	2.2743	0.0790
Gender × Region × Occupation	15.7904	2	20.5700	1.0643	0.3457
Gender × Age × Occupation	123.4202	6	8.3360	2.7729	0.0115*
Region × Age × Occupation	50.0161	6	11.3927	1.1237	0.3469
Gender × Region × Age × Occupation	56.9637	5	7.4182	1.5358	0.1768
Error	4176.4425	563			

*p < .05.

**Table 15:** Simple interaction effect of occupation and strengthening one’s immune system.

Source	SS	df	MS	F	Sig
**Government employee**					
Gender	12.6084	1	12.6084	1.5116	0.2202
Age	4.3392	3	1.4464	0.1734	0.9143
Gender × Age	101.6252	3	33.8751	4.0612	0.0078**
Error	1768.3136	212	8.3411		
**Healthcare**					
Gender	19.9446	1	19.9446	3.1276	0.0803
Age	20.0658	3	6.6886	1.0489	0.3748
Gender × Age	26.0612	3	8.6871	1.3623	0.2592
Error	586.6783	92	6.3769		
**Others**					
Gender	1.9862	1	1.9862	0.2784	0.5982
Age	16.6562	3	5.5521	0.7781	0.5071
Gender × Age	22.8729	3	7.6243	1.0685	0.3628
Error	2012.2813	282	7.1357		

**p < .01.

## Discussion

This study investigated Taiwanese citizen’s responses to COVID-19, including optimistic bias, social trust, information credibility, and protective measures adopted. Regarding protective measures, most people washed their hands frequently with soap as often as they could, which is consistent with findings from another study^33^. To strengthen their immune systems, people tried to drink more water (a traditional way approach^34^). People in Taiwan believe that water is the best medicine. In addition, when people felt ill, they would distance themselves from others, and many people avoided unnecessary travel. These behaviors reflect how people avoided contact with others as a simple precaution.

Social trust, information credibility, and personal protective measures were positively correlated, as were information credibility, avoiding human contact, and strengthening one’s immune system. When people trust the government and its information, they also take more precautionary behaviors. In addition, behaviors for avoiding human contact were positively correlated with behaviors for strengthening one’s immune system. This finding suggests that people who were highly focused on their behavior took more related precautions. However, optimistic bias and strengthening one’s immune system were negatively correlated, suggesting that the greater the disparity between perceived risk to self and to others, the less likely a person is to work on strengthening their immune system. A result could be that one might be overly optimistic and ignore the situation because of one’s healthier immune system. Accordingly, further analyses should be conducted on optimistic bias and the relationships among these key variables.

As the results of this study showed, optimistic bias existed in the population. Whether the self was compared to neighbors and colleagues or whether the self was compared to others, the differences were significant. Most individuals believed that they were less likely to be infected with COVID-19 than others were, which is a systematic error. This error was more obvious when the others were farther from the self. Members of the public tended to optimistically believe that they would not catch the disease. However, unlike previous research^14^, the results of this study showed no significant correlation between the optimistic bias and other personal protective measures. Shukla, et al.^35^ pointed out that an optimistic bias may lead to risky behavior and that therefore, it may still be effective to reduce optimistic bias in order to avoid public expose to risk.

The results on information credibility showed that Taiwan’s government is highly trusted. Of all the information from various sources—family, friends, newspapers, TV, the Internet, social media, academic institutes and government—the information provided by the government scored the highest in terms of credibility. When people sought information, they tended not to entirely believe unknown sources of online information, and tended to maintain trust in the government. This result is similar to that found for Korean people^36^. In the middle of February 2020, a survey conducted by the media also indicated that public satisfaction with the government was very high^37,38^. Although the public’s confidence in the government may have been prompted by the unknowns of the pandemic, Devine, et al. ^39^ suggested that trust in government may be due to compliance, or influenced by infectious cases, or even government policy. By reading professional or academic reports from research institutes or government agencies, people’s anxiety may be reduced^17^.On the other hand, if people pay too much attention to COVID-19 information, it may increase their anxiety^40^.Why Taiwanese people choose to trust their government needs further exploration.

In this study, four-way ANOVAs were conducted to explore the relationships among the demographic variables as independent variables and the six key dependent variables (i.e., optimistic bias, social trust, information credibility, personal protective measures, avoiding human contact, and strengthening one’s immune system). The four-way ANOVA of the optimistic bias showed a special trend in gender and age. Older males tended to be more optimistic, and older females tended to be less optimistic. The reasons for these opposite trends could be an interesting issue to study in the future (e.g., Sürücü, et al.^41^). Furthermore, people working in healthcare had less optimistic bias than did workers in other occupations. According to Weinstein^8^, especially for negative events, personal experience is the main factor influencing a person’s optimistic bias. Healthcare workers have experience with infectious diseases, and they might be less likely to be influenced by the bias. Individuals’ perceived risks of infection are different^42^ as demonstrated by the analysis of demographic variables.

Social trust interacted with gender and age, indicating opposite trends for people aged 41–50 years and people aged 51 years or more. Older males had higher social trust than older females, but younger males had lower social trust than younger females. Future research could explore other reasons for the relationship between trust of the government and COVID-19 in middle-aged people. However, no demographic differences in information credibility were found, indicating that different groups of people held similar attitudes toward the credibility of COVID-19 information.

To explore how different demographic variables can influence precautionary behaviors, three separate precautionary behaviors, namely, personal protective measures, avoiding human contact, and strengthening one’s immune system were examined. There were interactions of gender, region of residence, and occupation in the personal protective measures analyses. Especially for government employees, the gender difference in other regions of residence was larger than the gender difference in the Northern region. In both regions, males tended to take fewer personal protective measures than did females. Regional differences should be considered in policy promotion. Gender and occupation interacted. Especially for government employees and people in the other occupations category, males took fewer personal protective measures than did females. In general, males took fewer personal protective measures than females, and government employees and healthcare workers took more personal protective measures. The promotion of personal protective measures should focus greater attention on males and workers in other occupations.

In avoiding human contact, there were interactions of employees, gender, region and occupation. The results showed that males living in other regions engaged in fewer behaviors to avoid human contact, so this group might be at greater risk of infection and need further education about COVID-19. The interaction of gender and region showed different trends for males and females; males engaged in fewer such behaviors, as did individuals living in the northern region. In addition, the main effect of age also indicated that people under 30 years of age would not avoid contact with others, suggesting that the behaviors of young adults or teenagers might increase their risk relative to other groups during an outbreak of COVID-19. Of course, social distancing can also correspond to bad outcomes such as depression or anxiety^43^.There needs to be a balance between people’s mental and physical health^44^.

As for behaviors for strengthening one’s immune system, there was an interaction of gender, age, and occupation. For government employees, there was a significant simple interaction. Males and females engaged in different behaviors. The older a man was, the more behaviors he would engage in to strengthen his immune system.

Throughout all of these four-way ANOVA tests, the most important factor that influenced behavior was gender, and then occupation or region. Other factors were not statistically significant. For policy decisions, these can be considered non-priority factors. To ensure that all information concerning COVID-19 is properly and effectively disseminated, communication strategies accounting for differences in gender, occupation, and region of residence should be considered. However, the interaction effect of government employment were easily significant, indicating that they may have consensus on COVID-19. Given the relation of their jobs to government or schools, they can be regarded as a bridge between the public and the government, so their consensus and literacy on COVID-19 is very important. A question that remains is why these government employees did not have consistent attitudes and behaviors. This question should be further examined.

So far, the Central Epidemic Command Center has focused its communication policy on emphasizing strengthening personal immunity; as well as washing hands regularly; avoiding contact with the eyes, nose, and mouth; and taking other personal protective measures. It remains important to advise the public to take simple precautions against the spread of COVID-19 in the community. The center advised people not to go to hospitals when not necessary, and not to gather in crowds, and to practice social distancing. All of these factors in the social climate suggest that the public is being actively engaged in fighting the virus. However, additional outreach strategies still need to be considered because of the optimistic bias among some groups. To reduce the optimistic bias, more information should be provided. Studies have found that new media, such as simulation games, may be more useful or trustworthy than government outlets^42,43^. Other studies have also shown that people's stress is related to their use of media, which may increase trust in the local health care system^18^.

## Conclusions

The analysis showed that people in Taiwan are subject to the optimistic bias with respect to COVID-19 and thus may be overly optimistic in facing the epidemic. Accordingly, relevant information should be properly disclosed to help reduce the optimistic bias. However, because the respondents reported high social trust and endorsed the information credibility of government communication, Taiwanese citizens recognized COVID-19 as a serious problem. As a result, they implemented many precautionary measures to combat the new virus. Improving the relationship between the government and the public will improve communication. The government and the people cannot relax precautionary measures; everyone should fight COVID-19 by engaging in behaviors such as washing one’s hands until the virus is under control worldwide.

## Data Availability

The datasets used and analyzed during the current study are available from the corresponding author on reasonable request.
